# Identification of a New Target *slr0946* of the Response Regulator Sll0649 Involving Cadmium Tolerance in *Synechocystis* sp. PCC 6803

**DOI:** 10.3389/fmicb.2017.01582

**Published:** 2017-08-15

**Authors:** Tao Sun, Le Xu, Lina Wu, Zhongdi Song, Lei Chen, Weiwen Zhang

**Affiliations:** ^1^Laboratory of Synthetic Microbiology, School of Chemical Engineering and Technology, Tianjin University Tianjin, China; ^2^Key Laboratory of Systems Bioengineering, Ministry of Education of China Tianjin, China; ^3^Collaborative Innovation Center of Chemical Science and Engineering Tianjin, China; ^4^Center for Biosafety Research and Strategy, Tianjin University Tianjin, China

**Keywords:** cyanobacteria, cadmium tolerance, response regulator, Sll0649, Slr0946

## Abstract

Survival of photosynthetic cyanobacteria is challenged by environmental contaminations like heavy metals. Among them, deciphering the regulatory mechanisms for cadmium (Cd) in cyanobacteria would facilitate the construction of Cd-resistant strains. In this study, the DNA-Affinity-Purified-chromatin immunoprecipitation assay was employed to identify the direct targets of Sll0649, which was a Cd^2+^-related response regulator identified in our previous work in model cyanobacteria *Synechocystis* sp. PCC 6803. As a result, the promoter region of *slr0946* encoding the arsenate reductase was enriched fourfolds by quantitative real time PCR analysis. Further, deletion of *slr0946* led to a sensitive phenotype to Cd^2+^ stress compared with the wild type (WT) and the sensitive phenotype of Δ*slr0946* could be rescued by complementation assay via introducing *slr0946* back into Δ*slr0946*. Finally, individually overexpression of *slr0946* as well as two Cd^2+^-related genes identified priviously (i.e., *sll1598* and *slr0798*) in WT could significantly improve the tolerance of *Synechocystis* sp. PCC 6803 to Cd^2+^. This study provided a better understanding of the tolerance mechanism to Cd^2+^ in cyanobacteria and also feasible strategies for tolerance modifications to heavy metals in the future.

## Introduction

Photosynthetic cyanobacteria are a large group of Gram-negative prokaryotes able to utilize CO_2_ and sunlight directly for growth (Gao et al., 2016), playing a pivotal role in the global carbon and nitrogen cycling as well as in some bioremediation applications (Rahman et al., 2011). Notably, cyanobacteria have also been utilized as “photosynthetic microbial factories” and dozens of biofuels and chemicals have been successfully synthesized in recent years (Gao et al., 2016). Though various metals were required for growth, survival of cyanobacteria is challenged by heavy metals like arsenic (As), cadmium (Cd), mercury (Hg), and lead (Pb), which are increasingly spread out in the environment through human activities (Cassier-Chauvat and Chauvat, 2015). Among them, Cd^2+^ is toxic to cyanobacteria even at a low concentration thus it is important to investigate the response mechanisms of cyanobacteria to Cd^2+^ and then modified the tolerance to Cd^2+^. Sas et al. (2006) monitored the effect of Cd^2+^ on the photosynthetic activity of model cyanobacteria *Synechocystis* sp. PCC 6803 (hereafter *Synechocystis*), founding that Cd^2+^ could penetrate rapidly into the cells and blocked the photosynthetic activity by altering the whole-chain electron transport (Sas et al., 2006). In addition, Toth et al. (2012) claimed that the toxic effect of Cd^2+^ on *Synechocystis* could be a cascade mechanism, in which the primary effect involved the rapid inhibition of CO_2_-dependent electron transport while the secondary effect related with inhibitory influences on PS II electron transport as well as the degradation of the reaction center protein D1 (Toth et al., 2012). Nevertheless, the response mechanisms of cyanobacteria to Cd^2+^ were rarely elucidated (Chen et al., 2014b). A better understanding of the regulation mechanisms of cyanobacteria to Cd^2+^ would benefit the tolerance modifications of cyanobacteria in the future.

Two-component systems (TCSs) are important regulatory mechanisms allowing microorganisms to sense and respond to environmental changes and stress conditions (Los et al., 2010). Each of them contains a histidine kinase (HK) serving as a sensor to perceive a specific environmental stimulus and a corresponding response regulator (RR) to regulate the expression level of target genes (West and Stock, 2001). Besides the participation of TCSs in biological processes such as membrane porin regulation (Yuan et al., 2011) and cell communications (von Bodman et al., 2008), recent studies have found the crucial roles of TCSs in bacterial stress responses like ion stress (Los et al., 2010; Prabhakaran et al., 2016). For example, the ColRS operon composed of a HK ColS and a RR ColR was found related with Cd^2+^ and Mn^2+^ resistance as a lack of ColRS caused a five-fold reduction in resistance to Mn^2+^ in *Pseudomonas putida* CD2 (Hu and Zhao, 2007). In addition, the TCSs of CzcRS in *P. aeruginosa* and two regulatory systems (i.e., CusRS and CueR) in *Escherichia coli* were proved related with Zn^2+^ and Cu^2+^ stress response, respectively (Grass and Rensing, 2001; Caille et al., 2007). These studies suggested the important roles of TCSs in bacterial ion stress regulation and its potential application in tolerance modifications.

In *Synechocystis*, more than 90 genes were believed to encode a HK or RR protein (Gao et al., 2016). Among them, several proteins have been functionally characterized and proved to be related with various biological processes as well as abiotic stresses (Los et al., 2010; Liu et al., 2015). Our recent efforts using functional genomics strategies to study the metabolic responses of *Synechocystis* to various abiotic stresses also discovered several stress-responsive genes including a RR gene *slr1909* involving acid stress and two RR genes (i.e., *sll0039* and *slr1037*) directly related with 1-butanol stress (Chen et al., 2014a; Ren et al., 2014; Niu et al., 2015). Notably, our previous study also identified a RR gene *sll0649* involved in Cd^2+^ tolerance in *Synechocystis* (Chen et al., 2014b). Besides, *sll1598* and *slr0798* were proved to be the target of *sll0649* via electrophoretic mobility shift assays (EMSAs) (Chen et al., 2014b). In this study, to further explore the Cd^2+^ resistance mechanism in *Synechocystis*, DNA-affinity-purified chip (DAP-chip) assays was employed to identify new targets of Sll0649. The DAP-chip assay successfully identified another new target, i.e., *slr0946*, related with Cd^2+^ stress response. In addition, individually overexpression of all three targets of *sll0649* (i.e., *sll1598*, *slr0798*, and *slr0946*) could improve the tolerance of *Synechocystis* to Cd^2+^. Our work here provided new insights about the Cd^2+^ regulatory mechanisms in cyanobacteria and also feasible strategies for tolerance modifications to heavy metals.

## Materials and Methods

### Bacterial Culture Conditions

*Escherichia coli* BL21 (DE3) and *E. coli* DH5α were grown in the standard liquid LB medium or on agar plate with appropriate antibiotic (i.e., 10 μg/mL kanamycin) at 37°C using a shaking incubator at 130 rpm or incubator (Honour, Tianjin, China). Wild type *Synechocystis* (WT), mutants and the constructed strains were grown on agar plate or in BG11 medium at pH 7.5 using an illuming incubator or shaking incubator at a light intensity of approximately 50 μmol photons m^-2^s^-1^ and 130 rpm at 30°C (Honour, Tianjin, China). Medium for mutants and constructed strains was supplemented with appropriate antibiotic(s) (i.e., 10 μg/mL chloramphenicol and/or 10 μg/mL kanamycin). All strains and plasmids used in this study were listed in **Table 1**.

**Table 1:** Strains and plasmids used in this study.

Strain	Genotype‘	Reference
*E. coli* DH5α	F^-^, φ80d *lac*Z△M15, Δ(*lac*ZYA-*arg*F) U169, *deo*R, *rec*A1, *end*A1, *hsd*R17(r_k_^-^,m_k_^+^), *pho*A, *sup*E44, λ-, *thi*-1, *gyr*A96, *rel*A1	Stratagene
*E. coli* BL21	F^-^, *ompT gal dcm lon hsdS_B_*(r_B_^-^ m_B_^-^) *araB::T7RNAP-tetA*	Stratagene
*Synechocystis* sp. PCC 6803	WT	ATCC 27184
Δ*sll0649*-pJA0649	pJA2::PpsbA2-*sll0649*, Km^R^ in Δ*sll0649* strain	This study
Δ*slr0946*	Δ*slr0946*::Cm^R^	This study
Δ*slr0946*-pJA0946	pJA2::PpsbA2-*slr0946*, Km^R^ in Δ*slr0946* strain	This study
WT-pJA0649	pJA2::PpsbA2-*sll0649*, Km^R^ in WT	This study
WT-pJA0798	pJA2::PpsbA2-*slr0798*, Km^R^ in WT	This study
WT-pJA1598	pJA2::PpsbA2-*sll1598*, Km^R^ in WT	This study
WT-pJA0946	pJA2::PpsbA2-*slr0946*, Km^R^ in WT	This study
**Plasmids**		
pJA2		Huang et al., 2010; Kaczmarzyk et al., 2014


### Strains Construction

All primers used in this study were listed in Supplementary Table S1.

For gene deletion, the homologous recombination method was employed for the construction of gene knockout fragments for *slr0946* (Chen et al., 2014b). Briefly, the chloramphenicol resistance cassette (amplified from pACYC184), two flanking homologous arms (about 1 kb) were employed for overlapping PCR and replacing the target gene of *Synechocystis* by natural transformation. The successful knockout mutant was confirmed by PCR and purified via successive passages.

For gene complementation and overexpression, a replicative vector pJA2 kindly provided by Prof. Paul Hudson (KTH Royal Institute of Technology of Sweden) was employed to overexpress *sll0649*, *sll1598*, *slr0798*, and *slr0946*, respectively (Huang et al., 2010; Kaczmarzyk et al., 2014). The resulting plasmid pJA2-*sll0649* and pJA2-*slr0946* was, respectively, back introduced into Δ*sll0649* and Δ*slr0946*, leading to complementation strains Δ*sll0649-*pJA0649 and Δ*slr0946*-pJA0946. In addition, the resulting plasmid pJA2-*slr0946*, pJA2-*sll0649*, pJA2-*sll1598*, and pJA2-*slr0798* were, respectively, introduced into the WT, leading to the overexpression strains WT-pJA0946, WT-pJA0649, WT-pJA1598, and WT-pJA0798. The transformation was performed using GenePulser Xcell (Bio-Rad, Hercules, CA, United States) (Sun et al., 2017). The positive colonies were validated by PCR.

### Growth Patterns under Cd^2+^ Stress

For growth patterns, 5 mL fresh cells at OD_630 nm_ = 0.2 were collected by centrifugation (4°C, 3000 × *g* for 15 min) and then were inoculated into 25 mL BG11 liquid medium in a 100 mL flask with or without CdSO_4_, each with three replicates (the concentration of CdSO_4_ was 4.6 μM for WT and deletion mutants but 5.0 μM for WT and overexpression strains). Cell density was measured on an ELx808 Absorbance Microplate Reader (BioTek, Winooski, VT, United States) at OD_630_ (Sun et al., 2017). Growth experiments were repeated at least three times to confirm the phenotype.

### Overexpression and Purification of His_6_-Sll0649 Protein

Overexpression and purification of His_6_-Sll0649 protein were carried out as described previously (Chen et al., 2014b). Briefly, the *sll0649* gene was amplified and then cloned to pET46 Ek/LIC vector, resulting in the plasmid pET46-*sll0649*. The pET46-*sll0649* plasmid was then transformed into *E. coli* BL21 (DE3). The expression of His_6_-Sll0649 was induced by 0.1 mM isopropyl β-D-1-thiogalactopyranoside (IPTG) and followed by incubation at 22°C overnight. His_6_-Sll0649 was purified by the Ni-NTA agarose chromatography (GE healthcare, Uppsala, Sweden).

### DAP-Chip Assay

DNA-affinity-purified chip assays were employed to identify the genes that directly regulated by Sll0649. Promoter regions of 10 selected genes were amplified by PCR and incubated with the purified recombinant His_6_-Sll0649 to allow the possible enrichment after elution according to the protocols described in the literature (Rajeev et al., 2011). Briefly, the binding reactions (500 μL) were set up with 12 to 18 μg of sheared *Synechocystis* genomic DNA (with an average length of 500 bp) and purified protein in the incubation buffer [20 mM Tris-HCl, pH 7.5; 1 mM dithiothreitol (DTT); 5 mM MgCl_2_; 0.04 mg/mL BSA and 25% glycerol (v/v)]. The reactions were incubated at 25°C in a thermal cycler for 30 min; 50 μL of the reaction was then cleaned up by Qiaquick PCR purification columns (Qiagen, Hilden, Germany) and saved as input DNA. The rest was loaded to the Ni-NTA agarose chromatography that had been washed in the binding/wash buffer [20 mM Tris-HCl, pH 7.5; 10 mM MgCl_2_; 50 mM KCl; 25% glycerol (*v/v*)]. The enriched DNA was specifically eluted from the resin with 500 μL elution buffer [20 mM Tris-HCl, pH 7.5; 500 mM NaCl; 600 mM imidazole; 10% glycerol (*v/v*)]. The enriched DNA fractions were cleaned up and saved as output DNA. Input DNA and output DNA were quantified using the Nanodrop 2000 (Thermo, CA, United States).

### Quantitative Real Time PCR Analysis (qRT-PCR)

The qRT-PCR analysis was used to examine the enrichment fold of promoter regions of different genes after incubation with His_6_-Sll0649. Primers for qRT-PCR analysis were designed using Primer Express 2.0. To differentiate PCR products from primer dimers, primers were selected to generate amplicons with sizes around 100–200 bp. Experimental steps are based on the description previously (Sun et al., 2017). Three technical replicates were performed for each sample. Data analysis was carried out using the StepOnePlus analytical software (Applied Biosystems, Foster City, CA, United States) and the 2^-ΔΔC_T_^ method (Livak and Schmittgen, 2001). The *rnpB* gene encoding RNase P subunit B was used as an internal control (Chen et al., 2014a). Then the enrichment fold of output DNA was relatively quantified compared to that of input DNA. All primers were provided in Supplementary Table S1.

### Electrophoretic Mobility Shift Assay (EMSAs)

The EMSAs were performed as described previously (Chen et al., 2014b). Briefly, the promoter regions of *slr0946* and *slr1204* were amplified using the genomic DNA of *Synechocystis* and labeled with Cy5-labeled primer (5′-AGCCAGTGGCGATAAG-3′). The labeled PCR products were purified by QIAquick PCR Purification Kit (Qiagen, Hilden, Germany). In each EMSA reaction, ∼10 ng of Cy5-labeled DNA probes was incubated with varying amount of His_6_-Sll0649 protein in incubation buffer [1 mg/mL poly(dI–dC) (Roche, Basel, Switzerland), 20 mM Tris-base (pH 7.9), 1 mM DTT, 10 mM MgCl_2_, 0.2 mg/mL BSA and 5% glycerol (*v/v*)] for 20 min at 25°C. After incubation, protein-bound DNA and free DNA were separated by 6% Native-PAGE and viewed under Typhoon (GE healthcare, Uppsala, Sweden).

## Results

### Complementation of *sll0649* in Δ*sll0649*

**FIGURE 1:**
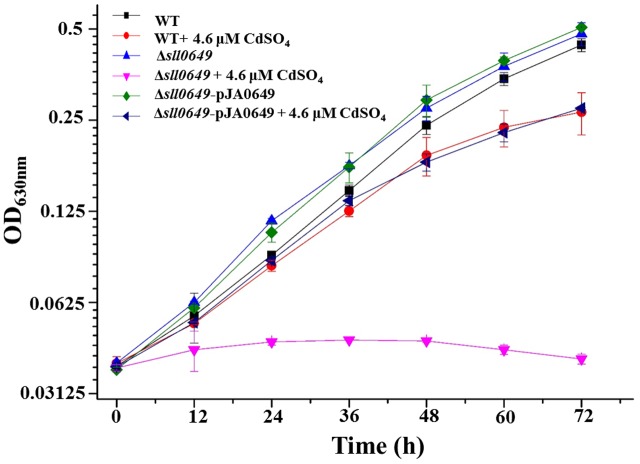
Growth patterns of WT, Δ*sll0649* and Δ*sll0649*-pJA0649 strains in BG11 medium with or without 4.6 μM cadmium sulfate. The error bars represented the calculated standard deviation of the three biological replicates.

In our previous work, a RR encoding gene *sll0649* was identified involved in Cd^2+^ stress response in *Synechocystis* and Δ*sll0649* exhibited more sensitive phenotype to Cd^2+^ than WT (Chen et al., 2014b). In this work, to further confirm the involvement of *sll0649* in Cd^2+^ tolerance, the *sll0649* gene was placed under the control of the *P*_psbA2_ promoter using a shuttle vector pJA2 and introduced back into the Δ*sll0649* mutant. The growth patterns among WT, Δ*sll0649* and Δ*sll0649*-pJA0649 strains were then tested under normal medium and medium with 4.6 μM Cd^2+^. As illustrated in **Figure 1**, no obvious growth differences were observed for all three strains under both normal BG11 medium. Under 4.6 μM Cd^2+^ stress condition, the complementation stain (i.e., Δ*sll0649*-pJA0649) was able to rescue the sensitive phenotype of the Δ*sll0649* to Cd^2+^, further suggesting the participation of *sll0649* in Cd^2+^ regulation (**Figure 1**).

### Identification of New Binding Targets of Sll0649 by DAP-Chip Assays

In our previous work, *sll1598* and *slr0798* have been identified as target genes of Sll0649 via EMSAs (Chen et al., 2014b). To further identify the new binding target(s) of Sll0649, DAP-chip strategy was employed. The full-length Sll0649 protein was first expressed in *E. coli* BL21(DE3) with a His_6_-tag at its N-termini. Extractive *Synechocystis* genomic DNA was sheared into 500–600 bp by sonification as input DNA. Purified His_6_-tagged Sll0649 proteins were incubated with sheared genomic DNA, and protein-bound DNA was purified using Ni-NTA resin to obtain the output DNA.

Then qRT-PCR was employed to determine the enrichment folds of different DNA regions. Ten candidate genes from upstream regions of *sll0649* were selected according to the previous results of quantitative proteomics analysis (Chen et al., 2014b). Among these ten candidates, seven of them (i.e., *sll0247*, *sll0248*, *slr0513*, *slr1204*, *slr0944*, *slr0945*, and *slr0946*) were found down-regulated in Δ*sll0649* compared to WT under Cd^2+^ stress, and the other three (i.e., *sll0041*, *sll0507*, and *sll0819*) were randomly selected as negative controls. The *rnpB* gene was used as a control for normalization in this study. The results of qRT-PCR were listed in **Table 2**, in which *slr0946* encoding the arsenate reductase was found enriched fourfolds among output Sll0649-bound DNAs compared to input DNA, suggesting it could be a new target of Sll0649.

**Table 2:** The results of quantitative real time PCR analysis (qRT-PCR).

Gene ID	Input *C*_t_ value	Output *C*_t_ value	2^-ΔΔ*C*_T_^
*sll0041*	25.118 ± 0.044	23.925 ± 0.490	1.229
*sll0507*	20.844 ± 0.065	19.654 ± 0.419	1.226
*sll0819*	21.897 ± 0.051	19.962 ± 0.025	2.055
*sll0247*	32.098 ± 0.199	30.943 ± 0.013	1.197
*sll0248*	32.031 ± 0.434	30.114 ± 0.007	2.029
*slr0513*	21.855 ± 0.076	20.202 ± 0.125	1.690
*slr1204*	22.526 ± 0.089	21.554 ± 0.145	1.054
*slr0944*	21.821 ± 0.011	20.756 ± 0.275	1.124
*slr0945*	15.004 ± 0.058	14.409 ± 0.066	0.812
*slr0946*	28.719 ± 0.107	25.741 ± 0.241	4.234
*rnpB*	18.713 ± 0.077	17.817 ± 0.063	


### Validation of Binding Target of Sll0649 through EMSAs

**FIGURE 2:**
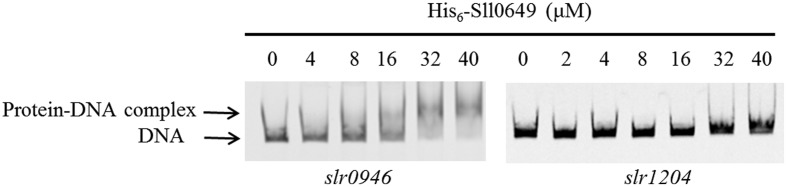
Electrophoretic mobility shift assays (EMSAs) to investigate the interaction of Sll0649 with promoter regions of *slr0946* and *slr1204*. The amounts of His_6_-Sll0649 (μM) used were as indicated and 10 ng each of 5′-cy5-labeled probes was added in the reaction of EMSAs.

In order to further verify the reliability of the new target *slr0946*, we performed EMSAs using purified His_6_-Sll0649 and the promoter region of *slr0946*. Meanwhile, the *slr1204* gene encoding degP was selected as the negative control. As shown in **Figure 2**, clear gel-shift pattern for the purified His_6_-Sll0649 with P*slr0946* was investigated while no direct binding was observed for the His_6_-Sll0649 with P*slr1204* under the testing condition, suggesting that Sll0649 was able to bind directly to the promoter region of *slr0946*.

### Functional Analysis of *slr0946* by Knockout and Complementation Assays

**FIGURE 3:**
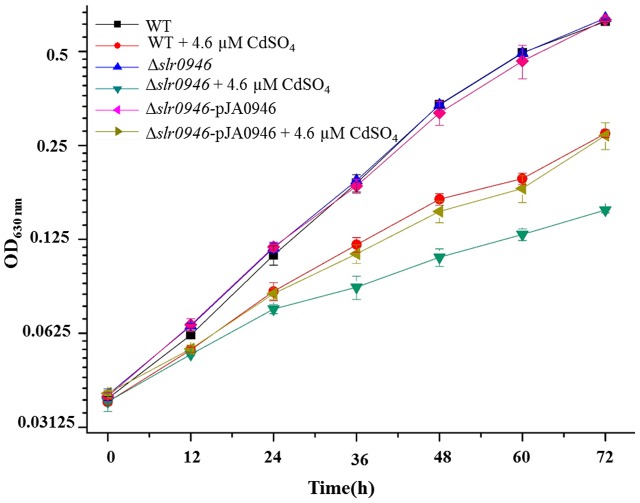
Growth patterns of WT, Δ*slr0946* and Δ*slr0946*-pJA0946 in BG11 medium with or without 4.6 μM cadmium sulfate. The error bars represented the calculated standard deviation of the three biological replicates.

To investigate the relationship of *slr0946* with Cd^2+^ stress response, knockout mutant was generated by inserting the chloramphenicol resistance cassettes to the opening reading frame (ORF) of *slr0946*. The Δ*slr0946* mutant was viable and its growth rate in the normal BG11 medium was similar to that of the WT (**Figure 3**). However, under 4.6 μM Cd^2+^ stress condition, Δ*slr0946* was found more sensitive to Cd^2+^ than WT (**Figure 3**), indicating its involvement in Cd^2+^ stress response. We further constructed a complementary mutant named Δ*slr0946*-pJA0946 by introducing the gene *slr0946* back into Δ*slr0946* using a shuttle vector pJA2. As expected, the Δ*slr0946*-pJA0946 strain was able to rescue the sensitive phenotype of Δ*slr0946* to Cd^2+^ in 4.6 μM Cd^2+^ stress (**Figure 3**), further confirming the participation of *slr0946* in Cd^2+^ stress response.

### Tolerance Modifications to Cd^2+^ via Overexpressing *sll0649*, *sll1598*, *slr0798*, and *slr0946*

Engineered Cd^2+^-resistant strains in *Synechocystis* could be promising and useful for further Cd^2+^ tolerance modifications in other cyanobacterial chassis. In this study, aiming to improve the Cd^2+^ resistance of *Synechocystis*, we respectively, overexpressed four genes related to Cd^2+^ resistance, i.e., *sll0649*, *sll1598*, *slr0798*, and *slr0946* in WT. The constructed strains were named as WT-pJA0649, WT-pJA1598, WT-pJA0798, and WT-pJA0946, respectively.

**FIGURE 4:**
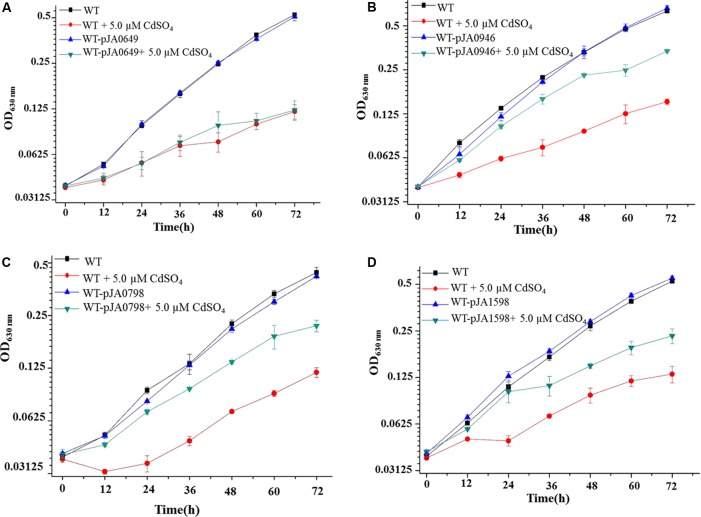
Growth patterns between WT and constructed strains. **(A)** Growth patterns between WT and WT-pJA0649 with or without 5.0 μM cadmium sulfate. **(B)** Growth patterns between WT and WT-pJA1598 with or without 5.0 μM cadmium sulfate. **(C)** Growth patterns between WT and WT-pJA0798 with or without 5.0 μM cadmium sulfate. **(D)** Growth patterns between WT and WT-pJA0946 with or without 5.0 μM cadmium sulfate. The error bars represented the calculated standard deviation of the three biological replicates.

Growth patterns showed no visible differences among all the four overexpression strains in the normal BG11 medium compared to WT (**Figure 4**). Excitingly, three of the four overexpression strains, i.e., WT-pJA1598, WT-pJA0798, and WT-pJA0946 had significant tolerance improvement compared to WT under 5.0 μM Cd^2+^ stress condition (**Figures 4B–D**). This indicated that overexpression of any of the three target genes of Sll0649 (i.e., *sll1598*, *slr0798*, and *slr0946*) could improve the tolerance of WT to Cd^2+^. However, we found that overexpression of *sll0649* can’t improve the tolerance of WT to Cd^2+^ due to some unknown reason (**Figure 4A**). To address this issue, the expression level of *sll0649* was measured by qRT-PCR in WT and WT-pJA0649. The result showed that the transcriptional level of *sll0649* gene in WT-pJA0649 was over 10-folds than that in WT (data not shown), suggesting that overexpressing *sll0649* gene can’t improve Cd^2+^ tolerance in *Synechocystis*.

## Discussion

It is well known that microbes tend to employ multiple resistance mechanisms in dealing with a single stress (Prabhakaran et al., 2016). Thus, it could be challenging to achieve tolerance improvement by sequentially engineering multiple genes. As manipulation of a regulatory gene might achieve simultaneous modifications of series of genes related to tolerance (Los et al., 2010), it has been proposed as an alternative strategy to focus on regulatory genes for tolerance modifications. In our previous study, a RR Sll0649 was found related with Cd^2+^ stress response (Chen et al., 2014b). In this study, complementation of *sll0649* in Δ*sll0649* rescued the sensitive phenotype though overexpression of *sll0649* can’t improve the tolerance of WT to Cd^2+^. As qRT-PCR showed overexpressed transcripts of *sll0649* in WT-pJA0649, we deduced that *sll0649* could control the Cd^2+^ response mechanism in *Synechocystis* but its expression level might already be saturated in WT.

Biochemical and/or genetic approaches have historically been used to study DNA-protein interactions. Among them, ChiP was a powerful and useful tool to obtain information of binding sites for RR. For example, by employing DAP-chip, Rajeev et al. (2011) presented a systematic experimental determination of the genes regulated by two RRs in *Desulfovibrio vulgaris* Hildenborough (Rajeev et al., 2011). In our DAP-chip assays along with qRT-PCR, Sll0649 was found to directly bind to the promoter region of *slr0946*. The *slr0946* gene appears to be located at the end of a gene cluster of *slr0944-slr0945-slr0946* belonging to the *arsBHC* operon. However, our results showed that there might be different regulation mechanisms for these three genes, since only the promoter region of *slr0946* was enriched (**Table 2**). Early studies have shown that the operon of *slr0944-slr0945-slr0946* was involved in arsenic sensing and resistance system in *Synechocystis* (Li et al., 2003; Lopez-Maury et al., 2003). In addition, *slr0946* encoding the arsenate reductase was found triggered by Cd in *Synechocystis* under the control of the regulator Slr1738 (Houot et al., 2007). In our previous study, Slr0946 was also among the down-regulated proteins in Δ*sll0649* after Cd^2+^ treatment (Chen et al., 2014b), which was consistent with the result that Slr0946 was triggered by Cd^2+^ stress (Houot et al., 2007). Notably, though overexpression of *sll0649* had no tolerance improvement of WT to Cd^2+^, overexpression of any of its three target genes including *slr0946*, *sll1598*, and *slr0798* could enhance the resistance to Cd^2+^, suggesting their relevance with Cd^2+^ stress response.

**FIGURE 5:**
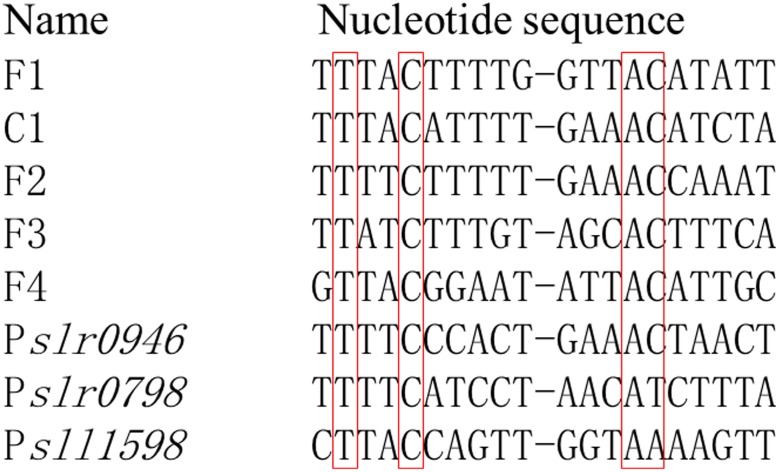
Potential conserved DNA-binding sequences for OmpR family RR Sll0649 existing in promoter regions of *slr0946*, *slr0798*, and *sll1598*. F1-F4 and C1 were reported previously as binding sequences for OmpR in *Escherichia coli* (Martinez-Hackert and Stock, 1997). Promoter regions for *slr0946*, *slr0798*, and *sll1598* were selected from the intergenic sequence between each target gene and its flanking gene. The conserved nucleotides were shown in red box.

In *E. coli*, OmpR serving as a transcriptional factor promoted the transcription of *ompF* in conditions of low osmolality while repressing the transcription of *ompF* and activating the transcription of *ompC* at high osmolality (Martinez-Hackert and Stock, 1997). In addition, the DNA binding sequences for OmpR have been elucidated, which were in a tandem arrangement and conserved bases were separated from each other by ten base pairs, or roughly one helical turn (Martinez-Hackert and Stock, 1997). For Sll0649 of *Synechocystis*, it shares a high identity up to 41% to OmpR of *E. coli* using Blastp^1^, suggesting the potential similarity for their target DNA sequences. Thus, we tried to find the potential conserved target sequences among the promoter regions of *slr0946*, *sll1598*, and *slr0798* according to the previous study (Martinez-Hackert and Stock, 1997). Interestingly, similar target sequences were found in all three genes (**Figure 5** and Supplementary Table S2), further suggesting their reliability as the targets of Sll0649. The Cd^2+^ stress could be sensed by Sll0649, then leading to the transcriptional activation of *slr0946*, *sll1598*, and *slr0798* related with stress response. In addition, Δ*sll0649* could hardly grew under 4.6 μM Cd^2+^ condition while growth of Δ*slr0946* was partially inhibited compared to Δ*sll0649*. This could be due to two possible reasons: (i) functional redundancy existed between *slr0946* and the other two targets thus partial function of *slr0946* could be replaced by *sll1598* and/or *slr0798*; (ii) the functional roles of *sll0649* was more important than that of *slr0946* as multiple genes could be activated by *sll0649* thus deletion of *sll0649* could cause a large deficiency of genes related with stress response.

In this study, a new target gene of the RR Sll0649, *slr0946*, was identified using DAP-chip and EMSAs. In addition, tolerance of *Synechocystis* was enhanced through overexpression any of the three target genes of Sll0649. This study deepened the tolerance mechanism of cyanobacteria to heavy metals and provided feasible strategies for tolerance modifications.

## Author Contributions

TS, LX, ZS, and LW performed the experiments. TS and LX wrote the manuscript. TS, LX, and LC analyzed the data. LC and WZ designed the study and revised the manuscript.

## Conflict of Interest Statement

The authors declare that the research was conducted in the absence of any commercial or financial relationships that could be construed as a potential conflict of interest.

## Footnotes

1 https://blast.ncbi.nlm.nih.gov/Blast.cgi
