# Supplementary material for: Identification of a New Target slr0946 of the Response Regulator Sll0649 Involving Cadmium Tolerance in Synechocystis sp. PCC 6803
Source: Front Microbiol. 2017 Aug 15;8:1582. doi: 10.3389/fmicb.2017.01582 (PMC5559466; doi:10.3389/fmicb.2017.01582)
Supplement: Supplementary file 1 [file Table_1.docx]

| **Table S1. Primers used in the present study** | |
| --- | --- |
| **Primer** | **Sequence (5’-3’)** |
| **1. Primers used in the process of protein overexpression** | |
| His_6_-Sll0649-F | GACGACGACAAGATGTGGGGGAACAGGACTGAA |
| His_6_-Sll0649-R | GAGGAGAAGCCCGGTTAATCAGGGTCTTCAAACTTATAGC |
| **2. The primers used for RT-PCR of target upstream regions** | |
| RT-*sll0041*-F | GTCGTCTGATGACTACTCCC |
| RT-*sll0041*-R | TCGGAGGAAGGATTTTGGGTC |
| RT-*sll0507*-F | ATCAACAAAGACACCGTCGC |
| RT-*sll0507*-R | TTCTTCTTGGGGATGACGCA |
| RT-*sll0819*-F | CCATTCTCTGCTAGTTTGAGT |
| RT-*sll0819*-R | AGGAGCAACGCCAACAAATG |
| RT-*sll0247*-F | TTACGTTACCCTTGGGCGAT |
| RT-*sll0247*-R | CCGAGAAACCTAGGTGGGATG |
| RT-*sll0248*-F | TGCAAACCTAGTCATTAGCCA |
| RT-*sll0248*-R | GTCCAATTTTTGTCATGATTACTGG |
| RT-*slr0513*-F | TCTCTTGCAATTATCCAGCAC |
| RT-*slr0513*-R | TCATCGAAGTCTAGGGCAATGG |
| RT-*slr1204*-F | TCAGATCCTAGACCCCGACTT |
| RT-*slr1204*-R | AAACGGCTTGGGCTGACATT |
| RT-*slr0944*-F | TGCGCCCCATCTTTAACAC |
| RT-*slr0944*-R | AGGGCAATGCCAATGACGA |
| RT-*slr0945*-F | CTTTGCTAGACCCCCGTTGT |
| RT-*slr0945*-R | CCGGGGAGGATGGTTAAAGT |
| RT-*slr0946*-F | CACGGCAACATTACAGGA |
| RT-*slr0946*-R | CGACAGGAATTACGTTTGC |
| RT-*sll0649*-F | CCCGCATTCGCCAACA |
| RT-*sll0649*-R | GGTCGATCTCCTCCCCTTTG |
| rnpB-F | GTGAGGACAGTGCCACAGAA |
| rnpB-R | GGCAGGAAAAAGACCAACCT |
| **3. Primers of upstream region of genes for EMSA** | |
| *slr1204*-F | AGCCAGTGGCGATAAGTCAGATCCTAGACCCCGACTT |
| *slr1204*-R | AGCCAGTGGCGATAAGAAACGGCTTGGGCTGACATT |
| *slr0946*-F | AGCCAGTGGCGATAAGCACGGCAACATTACAGGA |
| *slr0946*-R | AGCCAGTGGCGATAAGCGACAGGAATTACGTTTGC |
| **4. Primers used in constructing knockout strain** | |
| Cm-F2 | AGCTGATAGAAACAGAAGCCACTGG |
| Cm-R2 | TTACGCCCCGCCCTGCCACTCATCG |
| *slr0946*-F1 | CCAGTGATGTTAATGCTGGTGG |
| *slr0946*-R1 | CAGTGGCTTCTGTTTCTATCAGCTAGTTTTAGCCTCGCTTAGATTAATT |
| *slr0946*-F3 | GATGAGTGGCAGGGCGGGGCGTAAGATCTGCCCCATCCCCATTGATACC |
| *slr0946*-R3 | GCATCCCCGCCAAAATTACC |
| **5. Primers used in constructing and verifying pJA2-gene** | |
| pJA2-F | AGTAAGCCGGATCCACGCGT |
| pJA2-R | ATACACAGCCAGTCTCTAGAATAATTCC |
| pJA2-*sll0649*F | TGCTCTAGAATGTGGGGGAACAGGACTGAA |
| pJA2-*sll0649*R | CGCGGATCCTTAATCAGGGTCTTCAAACTT |
| pJA2-*sll1598*F | TGCTCTAGAATGGCAACATCATTTGCTTCCCGG |
| pJA2-*sll1598*R | CGCGGATCCTCATTGCTGGGCATTGGTACCGGC |
| pJA2-*slr0798*F | TGCTCTAGAATGACCCAATCTTCACCGCTCAAA |
| pJA2-*slr0798*R | CGCGGATCCTTACTTAGCAATCCGAGTAGCATT |
| pJA2-*slr0946*F | GCTCTAGAGCGTGAAAAAGGTAATGTTC |
| pJA2-*slr0946*R | CGGGATCCCGTCAGCTAATTTTGGCGATC |
| pJA2-511-F | GTTCCGCGCACATTTCCCCGA |
