# Supplementary material for: Identification of a New Target slr0946 of the Response Regulator Sll0649 Involving Cadmium Tolerance in Synechocystis sp. PCC 6803
Source: Front Microbiol. 2017 Aug 15;8:1582. doi: 10.3389/fmicb.2017.01582 (PMC5559466; doi:10.3389/fmicb.2017.01582)
Supplement: Supplementary file 2 [file Table_2.docx]

**Table S2．Potential promoters for *slr0946*, *slr0798* and *sll1598***

| Name | Sequence |
| --- | --- |
| P*slr0946* | tgtttttcccactgaaactaactccaatcactccaatttattccatcaattaattaatcaaactaattaatctaagcgaggctaaaact |
| P*slr0798* | agaggttggcgttaggagctagggaaaaatttaaactggatttagaaaatgattttcatcctaacatctttaatatctgagcatatcttcaggtgtttcaagatttgtgctacggttcaaggaggtttttctttaaatcacgttggccgcc |
| P*sll1598* | gtaaaaagcagaaagttttagtggtcagaaaaagcccacgagcttaccagttggtaaaagttaagaaattttaactccccgttttcactgcaattatgagaagataatgaaaaatgagatgatcatgaaagaccattatcact |
